# BRAF Non-V600 Mutations in Metastatic Colorectal Cancer

**DOI:** 10.3390/cancers15184604

**Published:** 2023-09-17

**Authors:** Junjia Liu, Hao Xie

**Affiliations:** 1 Albert Einstein College of Medicine, Bronx, NY 10461, USA; junjia.liu@einsteinmed.edu; 2 Department of Oncology, Mayo Clinic, Rochester, MN 55905, USA

**Keywords:** BRAF non-V600 mutation, colorectal cancer, targeted therapy, heterogeneity

## Abstract

**Simple Summary:**

Metastatic Colorectal cancer (CRC) is an aggressive and complex disease influenced by gene changes, including BRAF mutations. While previous work has largely spotlighted the BRAF V600 mutation, there remains much to uncover about its lesser-studied non-V600 counterparts. Through examining their characteristics, clinical relevance, and emerging treatment possibilities, this review bridges some knowledge gaps and paints a clearer picture of the BRAF non-V600 mutations. By understanding these intricacies, healthcare professionals and researchers can be better equipped to explore targeted treatments, potentially enhancing care for metastatic CRC patients.

**Abstract:**

Colorectal cancer (CRC) is the third leading cause of cancer-related deaths in the United States. Despite advancements in detection and therapeutic options, patients with metastatic CRC continue to face poor survival rates. The heterogeneity of oncogenic alterations, including BRAF mutations, poses a substantial challenge in identifying optimal treatment approaches. Notably, BRAF non-V600 mutations, encompassing class II and class III mutations, exhibit the distinct patterns of the signaling pathways and responses to targeted therapies compared to BRAF V600 mutations (class I). Nevertheless, the current classification system may underestimate the complexity and heterogeneity of BRAF-mutant CRC. Ongoing clinical trials are actively investigating targeted therapies for BRAF non-V600 mutations, but they are being confronted with patient recruitment obstacles due to the genetic diversity of these alterations. Continued research is needed to refine mutation subtyping, identify effective treatment strategies, and improve outcomes for patients with BRAF non-V600-mutant CRC. Enhancing our understanding and management of this specific subgroup of CRC is crucial for developing personalized treatment approaches and advancing patient care. This manuscript provides a comprehensive overview of the recent advances in and perspectives on BRAF non-V600 alterations in colorectal cancer, including relevant ongoing clinical trials.

## 1. Introduction

Colorectal cancer (CRC) is the third leading cause of cancer-related deaths in the United States [1]. Despite the recent advances in early detection using colonoscopy and stool DNA testing and in therapeutic options, the five-year overall survival (OS) of patients with metastatic CRC remains less than 16% [2]. Conventional chemotherapy regimens, which target rapidly dividing cells via impending DNA replication and causing DNA damage, yield a median survival of only 17–23 months for patients with metastatic CRC [3,4,5]. This can be much improved to approximately 36 months after the introduction of targeted therapy and immunotherapy [6]. However, one of the major challenges in finding optimal treatment for metastatic CRC is the heterogeneity of oncogenic mutations [7,8].

B-rapid accelerated fibrosarcoma (BRAF), a member of the rapid accelerated fibrosarcoma (RAF) kinase family, plays important roles in transducing growth signals in physiological processes [9,10]. However, mutant BRAF can result in the constitutive activation of the kinase cascade, leading to uncontrolled cell growth [9]. Notably, some well-studied BRAF mutations, such as the prevalent V600E genotype, have been identified as predictive biomarkers to the diminished response to irinotecan or oxaliplatin chemotherapy and as poor prognostic markers for patients with metastatic CRC [11,12]. Extensive research efforts have been devoted to exploring new treatment regimens for combating BRAF V600E mutations. These efforts have been comprehensively reviewed previously [13,14,15].

However, patient populations with BRAF mutations are heterogeneous [16,17]. Previous studies have identified more than 30 BRAF mutations associated with oncogenesis (Table 1). BRAF-mutant CRC, especially those with non-V600 mutations, can also have mutations in other BRAF-interacting proteins due to the complex nature of the MAPK pathway [18,19,20,21,22,23]. Metastatic CRC with BRAF non-V600 mutations may have distinct responses to targeted therapies compared to those with BRAF V600 mutations. The lack of effective agents targeting non-V600 genotypes and their rarity have hindered the further clinical evaluation of agents targeting BRAF non-V600 mutations and the establishment of treatment guidelines based on high-quality evidence. This review focuses on the recent advances in metastatic CRC with BRAF non-V600 mutations.

## 2. Classification of BRAF Mutations

**Figure 1:**
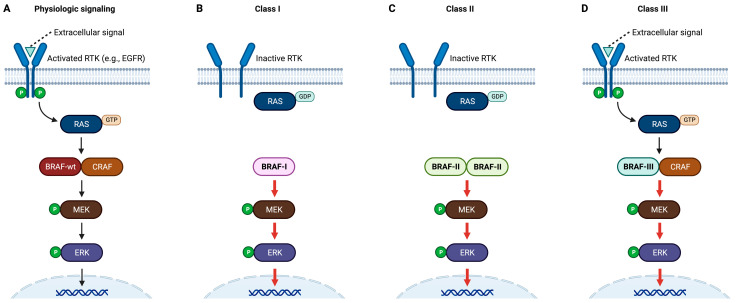
Simplified schemes of the RAS-RAF-MEK-ERK pathway. (**A**) Physiologic signaling: extracellular signal → RTK activation → RAS activation (from GDP-bound to GTP bound) → dimerization of autoinhibited RAF to an active dimer (e.g., heterodimerization of BRAF and CRAF) → MEK phosphorylation and activation → ERK phosphorylation and activation. Only a BRAF-wt/CRAF dimer is depicted here, but in physiologic processes, there are various RAS-dependent (upstream signal-dependent) dimers that are consistent with two proteins in the RAF family. (**B**) Class I: hyperactive BRAF with V600 mutation. No upstream signal is required, and the upstream components, such as RTK, may be inactivated due to negative feedback from the downstream. (**C**) Class II: hyperactive BRAF with mutations other than V600. The defining difference between class I and class II is the location of mutations (V600 versus non-V600) instead of the monomer/dimer status. (**D**) Class III: impaired kinase function on BRAF protein itself with paradoxically hyperactive downstream signaling. The enhanced downstream activity requires RAS signaling, which may be either transduced from further upstream or generated by constitutively activated mutant RAS. A representative illustration is depicted for physiologic signaling, class I, class II, and class III, respectively. However, readers should be aware that there are significant variations that still meet the inclusion criteria for each class. Created with BioRender.com. Abbreviations: BRAF, B-rapid accelerated fibrosarcoma (protein); BRAF-wt, wild-type BRAF (protein); BRAF-I, class I BRAF (protein); BRAF-II, class II BRAF (protein); BRAF-III, class III BRAF (protein); CRAF, B-rapid accelerated fibrosarcoma (protein); EGFR, epidermal growth factor receptor; ERK, extracellular signal-regulated kinase (also known as MAPK, mitogen-activated protein kinase); RAS, rat sarcoma (protein); GDP, guanosine-5′-diphosphate; GTP, guanosine-5′-triphosphate; MEK, mitogen-activated protein kinase kinase (also known as MAPKK); P, phosphorylated (activated); RTK, receptor tyrosine kinase.

Metastatic CRC has been classified by histology, sidedness, genetic alterations such as those in wingless-type MMTV integration site family member (wnt)/β-catenin, transforming growth factor beta/mothers against decapentaplegic homolog 4 (TGFβ/SMAD4), rat sarcoma (RAS), tumor protein 53 (TP53), mismatch repair pathways, and consensus molecular subtypes based on RNA expression profiles [27]. Among these aberrant signaling pathways, RAS signaling is the most well studied pathway in metastatic CRC. In normal physiology, the MAPK pathway is well regulated: upstream activation signal → RAS activation (from inactive GDP-bound to active GTP bound form) → dimerization of autoinhibited RAF to an active dimer (e.g., heterodimerization of BRAF and CRAF) → MEK phosphorylation and activation → ERK phosphorylation and activation. Oncogenic mutations in the BRAF gene are detected in approximately 10–12% of CRC cases, leading to uncontrolled signaling in the MAPK pathway (Figure 1) [16,28]. Among these mutations, V600 is the most common and, as a result, the most studied (typified as V600E), and it has been found to be associated with a poor prognosis in patients with metastatic CRC [14]. Studies, including clinical trials, showed that metastatic CRC with V600E mutations does not respond to anti-epidermal growth factor receptor (EGFR) antibodies compared to RAS wild-type CRC [29,30]. With the routine use of clinical next-generation sequencing platforms, more than 30 BRAF mutations were identified to be associated with malignancies [16,31]. Subsequently, various BRAF oncogenic mutations were discovered to elicit different responses to BRAF and/or MEK inhibitors [32]. Therefore, further classifications of BRAF mutations would be beneficial in guiding differential treatment strategies. Oncogenic BRAF mutations have been proposed to be classified into V600 mutations (class I) and non-V600 mutations, with the later further divided into class II and class III (Figure 1) [15,16,24,33].

In kinase-activated V600 mutations (class I), the mutant BRAF protein can mediate signal transduction with a constitutively active monomer in a RAS-independent manner. Class I mutations, particularly the prototype V600E, are the most extensively studied [14]. They account for 60–80% of BRAF mutations in CRC [16,34]. For class I mutations, signaling from the upstream receptor tyrosine kinase (RTK)/RAS is not needed due to the constitutive activation of monomeric BRAF (Figure 1B). BRAF V600-mutated monomers can be inhibited by vemurafenib, dabrafenib, or encorafenib. Notably, constitutively activated BRAF monomers, which do not require binding to another protein partner (such as CRAF) in the RAF family, are indicative of BRAF signaling in class I. Due to negative feedback, upstream RTK and RAS is suppressed. However, the presence of constitutively activated BRAF dimers or activated RAS does not preclude a mutation from being classified as class I. When there is high-level upstream RTK signaling, V600-mutated BRAF can form a dimer with a partner BRAF or CRAF molecule, resulting in a dimer resistant to anti-EGFR antibodies [35,36,37].

In class II, kinase-activated mutations occur outside the V600 site [35]. The possible locations of BRAF class II mutations include the protein activation segment and P-loop, as well as BRAF kinase domain fusion or duplication. Different from class I monomers, class II monomers are inactive, and they do not require upstream RAS signaling to form active dimers. In addition, CRAF is not required, as class II monomers can homodimerize to be able to activate downstream substrates. It has been found that first-generation BRAF inhibitors, including vemurafenib and dabrafenib, which preferentially inhibit the signaling of BRAF V600 monomers, are ineffective against BRAF dimers. The different responsiveness between BRAF monomers and BRAF dimers may involve a few mechanisms, including the negative cooperativity of the two binding sites on a BRAF dimer [38]. When a BRAF inhibitor binds to one site in the dimer, the affinity between the other site in the dimer and another inhibitor is reduced.

In class III, the BRAF mutation results in impaired kinase function with kinase activity from the BRAF protein itself, yet there is paradoxically increased signaling through the pathway [24]. Although the kinase activity of BRAF itself is impaired in class III, there are close interactions among RAS, BRAF, and another RAF partner (e.g., wild-type CRAF). There is increased binding between BRAF and CRAF, leading to the enhanced activation of CRAF, which results in increased downstream signaling. In class III, the dimerization between BRAF-mutated and wild-type CRAF requires signaling from upstream RAS. This RAS signaling may be either transduced from a source that is further upstream (e.g., RTK) or generated by a constitutively activated RAS mutant. For example, in CRC with BRAF class III mutations, concomitant RAS activation is typically due to signaling from the receptor tyrosine kinase (upstream to RAS). The same RAS activation mechanism occurs in BRAF class III lung cancers. However, dysregulated RAS, due to either RAS mutation itself or neurofibromin 1 (NF1) deletion/mutation, is the typical cause of RAS activation in BRAF class III melanomas. This difference means that BRAF class III CRC is supposed to be more sensitive to RTK (e.g., EGFR) blockade than BRAF class III melanomas [24]. In other words, combined targeted therapies involving upstream inhibitors might have different performances between different BRAF-mutated cancer types.

In summary, the primary inclusion criteria for class I are mutations located at V600 and BRAF kinase hyperactivation; for class II, they are mutations outside of V600 and BRAF kinase hyperactivation; and for class III, they are hypoactive BRAF and paradoxically hyperactive downstream signaling. There are also other types of mutations that do not fall into any of the above three classes (i.e., BRAF mutation of unknown significance) [14,15,23].

## 3. BRAF Non-V600 Mutations in CRC

Like other cancers, the management of BRAF-mutated metastatic CRC depends on various factors, including the aggressiveness and coexisting molecular alterations of the cancer, patient performance status and other medical comorbidities, and the goals of care [12]. Given the aggressiveness of BRAF-mutant metastatic CRC, folinic acid, 5-fluorouracil, oxaliplatin, and irinotecan (FOLFOXIRI) plus bevacizumab have been the mainstays of first-line therapy, especially in patients who are younger and have excellent performance status. Anti-EGFR antibodies such as cetuximab or panitumumab are generally not used in patients with BRAF mutations [12]. Upon disease progression, a combination of targeted agents on mutant BRAF should be utilized. Efforts to develop small molecule inhibitors against BRAF mutations have predominantly focused on V600 mutations. In CRC, the vertical inhibition of the MAPK pathway using a BRAF inhibitor such as encorafenib and EGFR blockade is the FDA-approved second-line therapy for patients with BRAF V600-mutated advanced CRC [39,40]. MEK inhibitors such as binimetinib are also effective, but their increased toxicities limit their application in this clinical setting [13]. However, these inhibitors might not be effective on CRC with BRAF class II or class III mutations due to their distinct signaling from class I mutations.

Some hypotheses have arisen to address the emerging questions on whether findings on BRAF class I mutant CRC are applicable to BRAF class II and class III mutant CRC. It is postulated that class II mutations may confer resistance to BRAF inhibitors like vemurafenib, dabrafenib, or encorafenib, as these agents are primarily effective against BRAF monomers and the constitutively activated BRAF in class II signals in the form of dimers [23,30]. Another hypothesis is that class III mutations that involve upstream signals from EGFR might exhibit greater sensitivity to EGFR inhibitors compared to class I or class II mutations [24]. Some retrospective studies have compared the features of cases with different BRAF classes. Patients with BRAF non-V600 CRC had distinct baseline demographic and clinical characteristics and distinct outcomes [37]. For example, compared to the V600E population, the non-V600 population exhibited a younger age (mean age: 58 versus 68, *p* < 0.001), a lower proportion of females (46% versus 65%, *p* < 0.001), right-sided localization (37% versus 82%, *p* < 0.001), high-grade tumors (13% versus 64%, *p* < 0.001), and a lower likelihood of microsatellite instability (MSI) (6% versus 13%, *p* < 0.001) while also demonstrating a better OS (hazard ratio: 0.18, *p* < 0.001) [37]. Regarding the difference of prognosis between BRAF class II and class III mutations, the reported findings have been more mixed. One study showed that both BRAF class I and class II mutations are associated with a worse prognosis compared to class III mutations, while another study observed similar survival outcomes in BRAF class II and class III mutations [32,37].

These disparate findings may reflect the complexity and variability within different subtypes of BRAF mutations and their impact on clinical outcomes. Further research is needed to understand the underlying factors contributing to these discrepancies. It is important to acknowledge that the current BRAF mutation classification system may underestimate the genetic complexity and heterogeneity of BRAF-mutated CRC. There are diverse mutations in the BRAF gene itself, and some BRAF mutations, particularly in class III, might be also associated with co-occurring mutations in other genes involved in the pathway [18,19,20,21,22]. The classification of BRAF mutations may need reevaluation and refinement to optimize its coherence and applicability in clinical settings.

Investigation on the functions of BRAF non-V600 mutations in CRC faces several challenges. Case studies examining the effectiveness of upstream inhibition on non-V600 mutations have had mixed results [24]. There is a need to identify distinct subgroups to tailor customized treatments. However, the patient population with BRAF non-V600 mutations is relatively small and encompasses diverse genetic alterations, making it challenging to design clinical trials for specific subgroups of the patient population. Without effective subcategorization, clinicians may underestimate the complexity of BRAF mutations and assume that their modes of oncogenic functionality or response to treatments (e.g., EGFR inhibitors) are similar [41]. Consequently, oncologists often hesitate to treat refractory class III patients with EGFR inhibitors, which target the upstream and are considered less effective in class I (V600 mutation) patients whose BRAF V600 mutation-driven hyperactivity is EGRF/RAS independent despite the dependency of class III mutations on upstream signaling [14].

Another obstacle in adopting novel treatment strategies in CRC is the divergent characteristics and drug responsiveness of BRAF-mutated cancers from different organ origins. For example, patients with BRAF class I mutations in melanoma and non-small-cell lung cancer exhibit good responsiveness to BRAF inhibitor [42,43]. However, the therapeutic activities on the same BRAF class I mutations in CRC patients are limited, likely caused by a rapid feedback activation of the upstream RTK and the resultant restoration of uncontrolled signaling in the pathway, a phenomenon which is specifically observed in CRC with BRAF mutations [44,45,46]. In other words, findings from clinical trials on BRAF mutations in other cancer types may not be transferrable to BRAF-mutant CRC.

Although non-V600 mutations are less common than V600 mutations and less studied, they are still present in a considerable portion of patients with BRAF mutations (21.6%) [37]. Non-V600 mutations encompass various alternations, but certain specific mutations, such as D594 mutations, are predominantly represented [37]. D594 mutations fall into the category of class III, which is hypothesized to have a more favorable prognosis and potential responsive to targeted therapies in retrospective analyses [24]. Further research on these mutation types may potentially extend survival and quality of life for a considerable number of patients.

## 4. Ongoing Clinical Trials in BRAF Non-V600-Mutant CRC

There are only a limited number of early phase clinical trials involving patients with BRAF non-V600-mutant CRC. These trials are often designed as “umbrella” or “basket” trials for all advanced solid tumors instead of being dedicated to patients with metastatic CRC. Without an adequate statistical power for comparison and the distinct biology of BRAF-mutant CRC compared to other cancer types, the findings from these trials that pertain to CRC are likely only hypothesis-generating. Nonetheless, findings from these studies can still be very valuable and serve to shine some light on the management of patients with BRAF non-V600-mutant CRC. Additionally, these trials are ongoing phase I or phase II trials with a single treatment arm assignment. Although they are well designed and studied as phase I or II trials with adequate statistical power to the proposed endpoints, their final patient enrollment and/or data analyses are not yet available for public scrutiny. As a result, early findings from these studies are mainly considered to be hypothesis-generating, especially with respect to preliminary activity signals in CRC, which will require further confirmation in larger clinical trials in the future.

Given the proven activity of the combination of BRAF and MEK inhibitors in BRAF V600-mutant tumors, the combination of encorafenib and binimetinib was tested in the phase II BEAVER trial (NCT03839342), targeting BRAF non-V600E mutations [47] (Table 2). Patients with BRAF non-V600E mutations in advanced solid tumor and no prior exposure to BRAF/MEK inhibitors received encorafenib (450 mg PO daily) and binimetinib (45 mg PO BID) on a 28-day cycle until intolerable toxicity or progression. The primary endpoint was overall response rate (ORR). Secondary endpoints included safety, disease control rate (DCR), progression-free survival (PFS), and OS. Two of the nine patients enrolled in the first part of this study had advanced CRC. Among the nine patients, one patient had a class I BRAF mutations, three patients had class II BRAF mutations, and five patients had class III BRAF mutations. One patient (12.5%) with BRAF G469S melanoma had unconfirmed partial response (PR), and one patient (12.5%) with BRAF D594N gallbladder cancer had stable disease (SD). Patient-derived xenograft models (PDXs) identified potential primary resistance mechanisms to encorafenib + binimetinib (EGFR and PI3K pathway activation and NF1 and RB1 loss of function) [47,48]. In addition, another phase I/II study (NCT03843775) with a similar design aiming to evaluate the safety and activity of encorafenib + binimetinib in patients with activating non-V600 BRAF mutant tumors is ongoing [49].

Trametinib (also known as Mekinist) dimethyl sulfoxide is an allosteric inhibitor of MEK1 and MEK2 proteins [50]. Trametinib, either alone or in combination with dabrafenib, has been approved for the treatment of cancer patients with BRAF V600E mutation. In addition to combination therapy, single agent trametinib was evaluated in the National Cancer Institute (NCI) MATCH trial subprotocol R (NCT04439279) for its activity in patients with BRAF fusion or non-V600E/K mutations who have not previously received MEK inhibitors [51]. In this study, trametinib dimethyl sulfoxide was administrated orally daily on a 28-day cycle. The primary endpoint was ORR. The secondary endpoints included six-month PFS rate. Thirty-three patients participated this study, and one patient responded to the trametinib. The six-month PFS rate was 20% (90% CI: 10–33%). PFS was 1.8 months (90% CI: 1.6–3.4 months) [51].

Inhibitors of ERK, downstream to RAF and MEK, have also been evaluated in patients with BRAF non-V600-mutant advance solid tumors for their safety and single-agent activity. ASN007 is a potent ERK1/2 inhibitor with an IC_50_ value of 1–2 nM. Group 6 of a phase I trial of ASN007 (NCT03415126) included patients with BRAF fusion or non-V600-mutant advanced solid tumors who had not previously received BRAF, MEK, or ERK inhibitors [52]. This study aimed to evaluate its safety, tolerability, pharmacokinetics (PK), and pharmacodynamics (PD) and sought to determine the maximum tolerated dose (MTD) in addition to its clinical activity. Forty-two patients were enrolled at various dose levels with MTDs at 40 mg daily and 250 mg weekly and dose limiting toxicities (DLTs) including grade-three central serous retinopathy, rash, and AST elevation. Stable disease in BRAF V600E mutant thyroid cancer was observed and lasted for more than 8 months [52,53]. Ulixertinib is another ERK1/2 inhibitor with anti-tumor activity in patients with BRAF non-V600 or MEK1/2 mutant tumors [54]. FDA rendered ulixertinib with fast-track designation for patients with BRAF (G469A, L485W, or L597Q) mutant solid tumors other than CRC. The phase II BVD-523-ABC trial (NCT04488003) aimed to assess the safety, PK, and PD of ulixertinib in patients with BRAF non-V600 and MEK mutant advanced malignancies who received 600 mg ulixertinib BID on a 28-day cycle. In part A of this trial, group 4 was dedicated to enrolling patients with BRAF non-V600 alterations. The primary endpoint was ORR; the secondary endpoints were duration of response (DOR), PFS, and OS [55,56].

The next-generation pan-RAF inhibitors have also been evaluated in phase I trials. In contrast to first-generation BRAF inhibitors, which are largely ineffective in patients with class II or III BRAF alterations, exarafenib (KIN-2787) is a potent and selective pan-RAF inhibitor that is specifically designed to inhibit class II and III BRAF dimers. Exarafenib inhibits RAF1, BRAF, and ARAF and has an IC_50_ value of 0.06–3.46 nM. Class II and III BRAF mutant cell lines were the most responsive to exarafenib, with IC_50_ values less than 50 nM. This anti-tumor activity was translated into in vivo activities in a dose-dependent manner in class II and III BRAF mutant PDXs when administrated with daily exarafenib. Exarafenib induced significant in vivo PK responses with the suppression of pERK and transcriptional changes in cells and PDXs [57]. Exarafenib is currently being evaluated in a phase I/Ib study (NCT04913285) for its safety, tolerability, PK, and clinical activity in patients with BRAF class I, II, and III and/or NRAS mutant advanced solid tumors. In this study, exarafenib is administered orally twice daily in a 28-day cycle. Both single-agent treatment strategies and treatment strategies consisting of using exarafenib in combination with binimetinib will be assessed in the dose escalation phase [58]. Similarly, BDTX-4933 is a pan-RAF inhibitor that targets all classes of oncogenic BRAF alterations and constitutively active KRAS or NRAS mutations. It is currently being evaluated in a phase I dose escalation and expansion study (NCT05786924) for its safety and anti-tumor activity in patients with class I, II, and III BRAF mutant or KRAS/NRAS mutant advanced solid tumors, including colorectal cancer [59].

## 5. Conclusions

In summary, BRAF non-V600 mutations have distinct functions and mechanisms of oncogenesis from BRAF V600 mutations in patients with metastatic CRC. Some of these BRAF non-V600 mutations are associated with improved prognosis and are potentially targetable. However, advancing the understanding and treatment of BRAF non-V600-mutant CRC presents several challenges. The current classification system for BRAF mutations may need further refinement to effectively stratify mutation subtypes. The majority of the clinical studies that have been conducted so far have predominantly focused on BRAF V600 mutations in advanced CRC, leaving a gap in the knowledge on targeting BRAF non-V600 mutations. Although some ongoing early-phase clinical trials are including patients with BRAF non-V600 mutations, their capacity to provide robust and specific analysis for novel agents targeting BRAF non-V600 mutations in CRC is yet to be determined. Therefore, further research is required to overcome these challenges and enhance our understanding and management of BRAF non-V600-mutant CRC.

## Figures and Tables

**Table 1:** Occurrences of BRAF mutations observed in colorectal cancer cohorts. Specific BRAF alternations and their assigned classes are based on the reports by Rosen [24] and VanderWalde [25]. It should be noted that BRAF fusions, although typically classified as class II, are not included in this table, as the specific alternations of BRAF fusions were not listed in the reports. The occurrences of these BRAF alterations in the GENIE v13.1 Public Cohort and the MSK-IMPACT Cohort have been obtained from cBioPortal [26]. The GENIE v13.1 Public Cohort comprised a total of 15,482 samples, including 1812 samples with BRAF mutations. The MSK-IMPACT Cohort comprised a total of 1007 samples, including 128 samples with BRAF mutations.

Class	Site	Alterations	Occurrences in CRC Cohorts	
			GENIE	MSK-IMPACT
Class I	Activation segment	V600D		
		V600E	1228	75
		V600K		
		V600L	1	
		V600M		
		V600R		
Class II	Activation segment	L597Q	1	
		L597R	3	
		L597S		
		L597V		
		T599R		
		T599dup	4	3
		K601E	14	2
		K601N	5	
		K601Q		
		K601T		
	P-loop	G464A		
		G464E		
		G464R	2	
		G464V	2	
		G469A	16	3
		G469R	12	
		G469V	10	
		V471F	2	
	Miscellaneous	Q257R		
		I463S		
		L485F	2	
		L485_P490delinsY		
		N486_P490del		
		V487_P492delinsA		
		K499E		
		L505F		
		L505H		
		E586K		
		V600_K601delinsE	1	
		V600_K601delinsEN		
		V600_S605delinsEISRWR		
Class III	Activation segment	T599A		
	P-loop	G466A	1	
		G466E	6	
		G466R	4	
		G466V	8	2
		S467L	1	
		G469E	6	2
	DFG motif	D594A	3	
		D594E	2	
		D594G	92	6
		D594H		
		D594N	21	3
		D594V	3	1
		F595L	6	
		G596C	1	
		G596D		
		G596R	2	
	Catalytic loop	N581I	5	1
		N581K		
		N581S	10	2
		N581Y		
	Miscellaneous	D287H		
		V459L		
		K483E	3	

Abbreviations: BRAF, B-rapid accelerated fibrosarcoma; CRC, colorectal cancer; GENIE, AACR Project Genomics Evidence Neoplasia Information Exchange; MSKCC, Memorial Sloan Kettering Cancer Center; N/A, not available.

**Table 2:** Ongoing clinical trials involving BRAF non-V600-mutant CRC.

Clinical Trial Number	Inhibitor(s)		Target(s)	Clinical Phase	Sponsor
NCT03843775	Combination of	Encorafenib	BRAF	Phase I/II	MSKCC (in collaboration with Array BioPharma)
		Binimetinib	MEK		
NCT03839342	Combination of	Encorafenib	BRAF	Phase II	University Health Network, Toronto
		Binimetinib	MEK		
NCT04439279	Trametinib		MEK	Phase II	NCI
NCT04488003	Ulixertinib		ERK	Phase II	BioMed Valley Discoveries, Inc.
NCT03415126	ASN007		ERK	Phase I	Asana BioSciences
NCT04913285	Exarafenib		BRAF	Phase I	Kinnate Biopharma
NCT05786924	BDTX-4933		BRAF	Phase I	Black Diamond Therapeutics, Inc.

Abbreviations: BRAF, B-rapid accelerated fibrosarcoma; ERK, extracellular signal-regulated kinase (also known as MAPK, mitogen-activated protein kinase); MEK, mitogen-activated protein kinase kinase (also known as MAPKK); MSKCC, Memorial Sloan Kettering Cancer Center; NCI, National Cancer Institute.
